# On the Use and Abuse of Alcoholic Liquors in Health and Disease

**Published:** 1850-12

**Authors:** 


*On the use and abuse of Alcoholic Liquors in health and disease. Prize Essay.* By WILLIAM B. CARPENTER, M. D., F. R. S., F. G. S., etc., etc., etc. Philadelphia: Lea & Blanchard. 1850. 12 mo. pp. 204.

A discussion, by one of the first of living physologist, of the effects of alcoholic liquors, when taken under various circumstances, in a state of health, and of their applicability in the treatment of diseases, could hardly fail to possess interest for the medical reader. The discussion is of interest in a purely scientific point of view, as involving questions of hygiene and therapeutics; and, in these aspects, the principles of the use and abuse of alcoholic liquors are by no means so fully settled, as not to leave room for considerable differences of opinion and practice among practitioners. It is, however, the moral relations of the subject that preeminently commend it to the attention of Physicians. To consider it in these relations, it is true, does not fall strictly within the province of medical science But practitioners of medicine cannot divest themselves, if they would, of peculiar responsibilities, in view of these moral relations. Their position is such that they must almost necessarily exert, by their opinions, example, and modes of practice, an influence either for, or against the evils of intemperance. The abuse of alcoholic liquors, to a certain extent, is correlative to their use—the former is liable to become a consequence of the latter. The latter may be affected in no small degree, by medical authority, and, hence, either for good or evil, this authority must, indirectly affect also the former. If this be a just view of the case, and that it is so can not, as we think, but be sufficiently apparent, then it behooves medical practitioners to study well the hygienic and therapeutic properties of alcohol, so that while they appreciate whatever importance it may possess, as a prophylactic or remedial agent, they may be prepared to discharge with fidelity the duties incident to the relations of their profession to the evils of intemperance. The responsibility is two-fold. We have no right to disparage alcoholic liquors as a means of preserving health, or relieving disease; but the conscience shrinks from any agency in the moral consequences flowing from an over estimate of the efficacy which belongs to them in their physical effects. The grand point, obviously, is to determine with the utmost practicable correctness and precision, the effects of alcohol in health and disease, and to deduce therefrom, in connection with the results of experience, the rules which are to guide its administration as a medicinal agent. The duty of the practitioner is discharged when he has impartially arrived at opinions on these points, after due consideration, with the light of present physiological and pathological knowledge; and when, practically, he acts in conformity with his best judgment, under a proper sense of responsibility as respects both the moral and physical consequences of his professional conduct.

After a careful perusal of the essay by Dr. Carpenter, we commend it most heartily to our professional brethren. And we think that the majority of medical readers will concur in the opinion that the several questions are discussed candidly, as well as ably, and that the conclusions of the article, in the main, are abundantly shown to be in accordance with established principles, in the present condition of medical science.

We quote from the preface the following propositions, embodying the conclusions just referred to:—

In the *first* place.—That, from a scientific examination of the *modus operandi* of Alchohol upon the human body, when taken in a *poisonous* dose, or to such an extent as to produce Intoxication, we may fairly draw inferences with regard to the specific effects which it is likely to produce, when repeatedly taken in excess, but not to an immediately fatal amount.

*Secondly.*—That the consequences of the *excessive* use of Alcoholic liquors, as proved by the experience of the Medical Profession, and universally admitted by medical writers, being precisely such as the study of its effects in poisonous and immediately fatal doses would lead us to anticipate, we are further justified in expressing that the habitual use of smaller quantities of these liquors, if sufficiently prolonged, will ultimately be attended, in a large proportion of cases, with consequences prejudicial to the human system,—the morbid actions thus engendered being more likely to be chronic, than acute, in their character.

*Thirdly.*—That as such morbid actions are actually found to be among the most common disorders of persons advanced in life, who have been in the habit of taking a "moderate" allowance of alcoholic liquors, there is very strong ground for regarding them as in a great degree dependent upon the asserted cause; although the long postponement of their effects may render it impossible to *demonstrate* the existence of such a connection.

*Fourthly.*—That the preceding conclusion is fully borne out by the proved results of the "moderate" use of Alcoholic liquors, in producing a marked liability to the acute forms of similar diseases in hot climates, where their action is accelerated by other conditions; and also by the analogous facts now univerally admitted, in regard to the remotely injurious effects of slight excess in diet, imperfect aeration of the blood, insufficient repose, and other like violations of the Laws of Health, when habitually practiced through a long period of time.

*Fifthly.*—That the capacity of the healthy Human system to sustain as much bodily or mental labor as it can be legitimately called upon to perform, and its power of resisting the extremes of Heat and Cold, as well as other depressing agencies, are not augmented by the use of alcoholic liquors; but that, on the other hand, their use, under such circumstances, tends positively to the impairment of that capacity.

*Sixthly.*—That, where there is a deficiency of power, on the part of the system, to carry on its nomal actions with the energy and regularity which constitute health, such power can rarely be imparted by the habitual use of alcoholic liquors; its deficiency being generally consequent upon some habitual departure from the laws of health, for which the use of alcoholic liquors compensate; and the employment of such liquors, although with the temporary effect of palliating the disorder, having not merely a remotely injurious effects *per se*, but also tending to mask the action of other morbific causes, by rendering the system more tolerant of them.

*Seventhly.*—That, consequently, it is the duty of the Medical Practioner to discourage as much as possible the *habitual* use of alcoholic liquors, in however "moderate" a quantity, by all persons in ordinary health; and to seek to remedy those slight departures from health which result from the "wear and tear" of active life by the means which shall most directly remove or antagonize their causes, instead of by such as simply palliate their effect.

*Eighthly.*—That, whilst the habitual use of alcoholic liquors, even in the most "moderate" amount, is likely (except in a few rare instances) to be rather injurious than beneficial, great benefit may be derived, in the treatment of diseases, from the *medical* use of Alcohol in appropriate cases; but that the same care should be employed in the discriminating selection of those cases, as would be taken by the conscientious practitioner in regard to the administration of any other powerful remedy which is poisonous in large doses.

The foregoing appear to the Author to be the conclusions legitimately deducible from the facts and arguments which he has brought forward; it will be for his Professional readers to decide how far the case which he has made out is sufficiently strong to lead them to the same results. This much, however, he would add: that, when he first entered upon the investigation, some years ago, he had adopted no foregone conclusion, and had, consequently, no temptation to make the facts square with preconceived views; that he has constantly endeavored to treat the subject as one of purely scientific inquiry, and has avoided mixing up any other considerations with those which presented themselves to him as a Physiologist and a Physician; and that, for the sake of keeping himself free from even the appearance of partisanship, he has never allied himself with any one of the Societies which have been formed to carry into practical effect the Total Abstinence principle, but has preferred to follow a perfectly independent course. He ventures to hope that on these grounds he may claim some right to being candidly heard by those to whom this Essay is more especially addressed.

For sale by Phinney & Co., and Derby & Co.

